# Male reproductive health after 3 months from SARS-CoV-2 infection: a multicentric study

**DOI:** 10.1007/s40618-022-01887-3

**Published:** 2022-08-09

**Authors:** D. Paoli, F. Pallotti, A. Anzuini, S. Bianchini, L. Caponecchia, A. Carraro, M. R. Ciardi, F. Faja, C. Fiori, D. Gianfrilli, A. Lenzi, M. Lichtner, I. Marcucci, C. M. Mastroianni, G. Nigro, P. Pasculli, C. Pozza, F. Rizzo, P. Salacone, A. Sebastianelli, F. Lombardo

**Affiliations:** 1 Laboratory of Seminology–Sperm Bank “Loredana Gandini”, Department of Experimental Medicine, “Sapienza” University of Rome, Viale del Policlinico 155, 00161 Rome, Italy; 2 Hormone Laboratory, Department of Experimental Medicine, “Sapienza” University of Rome, Rome, Italy; 3 Andrology and Pathophysiology of Reproduction Unit, Santa Maria Goretti Hospital, Latina, Italy; 4 Infectious Diseases Unit, Santa Maria Goretti Hospital, Sapienza University of Rome, Latina, Italy; 5 Department of Public Health and Infectious Diseases, “Sapienza” University of Rome, Policlinico Umberto I Hospital, Rome, Italy; 6 Department of Experimental Medicine, Sapienza University of Rome, Rome, Italy

**Keywords:** SARS-CoV-2, COVID-19, Sperm parameters, Anti-sperm antibodies, Sperm DNA fragmentation, Testosterone

## Abstract

**Purpose:**

While SARS-CoV-2 infection appears not to be clinically evident in the testes, indirect inflammatory effects and fever may impair testicular function. To date, few long-term data of semen parameters impairment after recovery and comprehensive andrological evaluation of recovered patients has been published. The purpose of this study was to investigate whether SARS-CoV-2 infection affect male reproductive health.

**Methods:**

Eighty patients were recruited three months after COVID-19 recovery. They performed physical examination, testicular ultrasound, semen analysis, sperm DNA integrity evaluation (TUNEL), anti-sperm antibodies (ASA) testing, sex hormone profile evaluation (Total testosterone, LH, FSH). In addition, all patients were administered International Index of Erectile Function questionnaire (IIEF-15). Sperm parameters were compared with two age-matched healthy pre-COVID-19 control groups of normozoospermic (CTR1) and primary infertile (CTR2) subjects.

**Results:**

Median values of *semen parameters* from recovered SARS-CoV-2 subjects were within WHO 2010 fifth percentile. Mean percentage of *sperm DNA fragmentation* (%SDF) was 14.1 ± 7.0%. Gelatin Agglutination Test (*GAT*) was positive in 3.9% of blood serum samples, but no positive semen plasma sample was found. Only five subjects (6.2%) had total *testosterone levels* below the laboratory reference range. Mean *bilateral testicular volume* was 31.5 ± 9.6 ml. *Erectile dysfunction* was detected in 30% of subjects.

**Conclusion:**

Our data remark that COVID-19 does not seem to cause direct damage to the testicular function, while indirect damage appears to be transient. It is possible to counsel infertile couples to postpone the research of parenthood or ART procedures around three months after recovery from the infection.

**Supplementary Information:**

The online version contains supplementary material available at 10.1007/s40618-022-01887-3.

## Introduction

Epidemiological data have identified male gender as a risk factor for severe COVID-19 and increased mortality [1]. This inequality is likely due to mixture of behavioral/lifestyle patterns, gender-specific incidence of comorbidities, aging and intrinsic biological differences between the sexes [1] either on hormonal (i.e., the different effects of testosterone, oestrogens or progesterone) or genetical basis. For this reason, the SARS-CoV-2 outbreak has induced researchers to focus on two substantial male reproductive health issues: first, the possible presence of SARS-CoV-2 in seminal fluid and, second, its impact on testicular function. ACE2, the molecular target for SARS-CoV-2, is expressed in the human testis but its association with testicular infection and, consequently, with impaired spermatogenesis is controversial. Several authors have investigated the presence of SARS-CoV-2 in semen but to date the main consensus is that the chance of detection in this biological sample is negligible: collectively, published data indicate that SARS-CoV-2 has been reported in around 3% (12/386) of investigated semen samples [2]. We could then conclude that the infection appears not to be clinically evident in the testes, although SARS-CoV-2 could theoretically reach semen from the blood, through the blood–testis barrier. This can be explained by the fact that ACE2 and TMPRSS2 are not co-expressed in testicular tissue [3]. Furthermore, to date, few studies have found the presence of SARS-CoV-2 in blood [2]. It is possible that the protection of the blood–testis barrier combined to the absent/low level of viremia in COVID-19 patients might contribute to the SARS-CoV-2 absence in the testis [4]. Besides, testicular damage may be indirectly caused by COVID-19 disease and spermatogenesis may be subsequently impaired through different mechanisms.

Infection and inflammation of the reproductive tract are important factors of infertility [5]. Severe COVID-19 induce high serum levels of pro-inflammatory cytokines and in critical cases it induces the so-called “cytokine storm” with high likelihood of tissue damage [6]. Several studies have shown high levels of seminal pro- and anti-inflammatory cytokines [7, 8] markers of apoptosis and impaired antioxidant activity along with compromised spermatogenesis in male patients recovering from COVID-19 [7], suggesting the presence of an inflammatory condition in the male genital tract. Dysregulated cytokines and chemokines may trigger an autoimmune reaction with consequent alteration of the testicular tissue [9]; TNF-α and IL-1β, in particular, may induce oxidative stress in the Sertoli cells and compromise the blood–testis barrier integrity [10]. This may trigger an anti-sperm autoimmune response, capable of affecting both semen quality (sperm concentration, motility) and the fertilizing ability of spermatozoa and the fusion of the gametes [11, 12]. Pro-inflammatory IL-6 could be involved in the alteration of Leydig cell differentiation as increased LH levels and decreased T/LH and FSH/LH ratios were found in COVID-19 patients compared to healthy [13]. Additional data suggestive of a SARS-CoV-2 interference with gonadal function are consistent with low Testosterone levels in COVID-19 patients [14, 15]. It could, therefore, be hypothesized that an alteration in semen quality could be caused by dysregulated inflammatory mediators, seminal antioxidant defense system and gonadal hormone levels [7]. Fever may also induce changes in testicular temperature that can also negatively impact on germ cells development [16] and transiently affect semen quality and sperm DNA integrity [17, 18]. Therefore, fever induced by COVID-19 can alter sperm parameters even in the absence of the virus in the semen [19]. Several studies evaluated the effect of SARS CoV 2 on semen quality showing impaired semen parameters. It is noteworthy that most of these studies performed semen analyses after a median of 30–40 days from the recovery [7, 20–22]. Since a spermatogenetic cycle takes approximately 78 days to be completed, it seems appropriate to evaluate semen quality at least 3 months from the recovery. However, few studies have considered semen analyses at least three months after recovery; moreover, these studies are characterized by small caseloads, contrasting results and even fewer considered testicular functional features other than semen analysis [13, 23–27].

For these reasons, we aimed to perform a comprehensive evaluation of the male reproductive health of COVID-19 patients after 3 months from the recovery, investigating:

- testicular function evaluating both semen parameters and hormone profile
- molecular aspects of spermatozoa by evaluation of DNA integrity
- the integrity of the blood–testis barrier analyzing the presence of anti-sperm antibodies.
- testicular morphological features through ultrasound evaluation
- sexual functioning through the self-administered questionnaire IIEF-15

## Materials and methods

### Patients

The study was approved by the Ethics Committee “Sapienza” (Prot. 0282/2021). Written informed consent was obtained from all study participants. Patients with previous SARS-CoV-2 infection occurred between July 2020 and January 2021 (before the opening of the Italian vaccination campaign to the whole population) were recruited in two Departments of Infectious Disease: AOU Policlinico Umberto I Hospital—“Sapienza” University of Rome and Santa Maria Goretti Hospital Latina (ASL Latina). Each patient was asked to perform an andrological screening to evaluate possible COVID-19 consequences.

Patients were recruited according to the following criteria:

- previous nasopharyngeal swab positive for SARS-CoV-2 between July 2020 and January 2021;
- 3 months after disease recovery (first negative nasopharyngeal swab);
- age from 18 to 65 years.

Men with andrological and systemic diseases, Klinefelter’s syndrome and other chromosomal conditions, genetic syndromes, diabetes, hypogonadism (total testosterone below 8 nmol/l), neoplasms, or previous chemotherapy and/or radiotherapy treatments, recent urinary tract infections, clinically relevant varicocele (clinical grade III) or any other andrological condition known to affect semen parameters and sperm DNA integrity were excluded from the study. COVID-19 severity was classified according to the WHO classification (Mild, Moderate, Severe, Critical) [28]. Three months after recovery, patients performed physical examination, testicular ultrasound and were asked to provide a semen and a blood sample. Medical history and other relevant clinical and biochemical data were retrieved from medical records of the subjects.

### Control groups

Semen parameters of COVID19 recovered subjects were compared with healthy controls recruited between 2018 and 2019 (before the SARS-CoV-2 appearance). In particular, we retrospectively selected:

*Control Group 1 (CTR1)*—Healthy normozoospermic subjects with no andrological diseases who attended the Laboratory of Seminology, Sperm Bank “Loredana Gandini” Department of Experimental Medicine—Sapienza University of Rome before the COVID-19 outbreak (2018–2019) for a pre-conceptional screening and, therefore, none of the Control group 1 subjects had children prior of semen analysis.

*Control group 2 (CTR2)*—Patients with idiopathic infertility but otherwise healthy who attended the Laboratory of Seminology, Sperm Bank “Loredana Gandini” between 2018 and 2019 for semen analysis as a part of an andrological work-up for couple infertility, in the absence of a detectable male or female factor (their partner simultaneously attended the gynecological dept. of our hospital).

### Centralized assessment

The principal bias in a multicentre study is the interlaboratory difference in analysis evaluation. For this reason, we centralized at the Laboratory of Seminology- Sperm Bank—“Sapienza” University of Rome the assessment of several parameters: sperm morphology (May-Grünwald–Giemsa staining), hormone analyses (FSH, LH, Testosterone), antisperm antibodies (indirect tests), sperm DNA fragmentation (TUNEL assay). The laboratory is currently a recognized andrological and seminological training center, accredited by major national and international Scientific Societies.

### Semen analysis

Semen samples were collected by masturbation after 2–7 days’ abstinence. All samples were allowed to liquefy at 37 °C for 60 min and were then assessed according to WHO (2010) [29]. The following variables were taken into consideration: volume (ml), total sperm number (*n* × 106 per ejaculate), progressive motility (%), and morphology (% abnormal forms). A sperm viability test was carried out to differentiate cell death from immotility by staining with eosin Y 0.5% in saline solution. As semen analysis was performed in two different Andrology Centres (Rome and Latina), standardization of analyses was achieved by the participation of each Centre in external quality control (EQC) programs and the execution of a routinary internal quality control (IQC).

### Antisperm antibodies (ASA) detection

*Direct ASA test*—Autoimmune reaction was evaluated on the sperm surface by the SpermMar test (FertiPro, Belgium) (WHO 2010). Direct tests could not be performed in hypokinetic or oligozoospermic samples. Light microscopy at 400 × was used to evaluate the percentage of motile sperm that presented latex particles (coated with human IgG or IgA) bound and the site of the bond (head, midpiece, tail).

*Indirect ASA test*—Autoimmune reaction was evaluated in blood serum and seminal plasma by the Gelatin Agglutination Test (GAT) [30]. All indirect tests were performed twice with different antigens.

Positivity was defined as a direct SpermMar test showing binding > 20%, but clinical relevance was considered with a binding percentage of > 50%. For indirect tests (GAT), an antibody titer of 1:32 or more in blood serum was considered clinically significant (1:16 in seminal plasma) [11].

### Sperm DNA fragmentation (SDF)

SDF was evaluated using TUNEL assay (Roche, In Situ Cell Death Detection Kit, Fluorescein, Roche, Basel, Switzerland). After assessment of semen parameters, the samples were centrifuged and evaluated as previously described by [31]. The samples were then analysed under fluorescent microscope (Leica DMR; Leica, Wetzlar, Germany), counting at least 500 cells.

### Hormone evaluation

Recruited subjects provided a peripheral blood sample at around 8 a.m. after overnight fasting. Serum follicle-stimulating hormone (FSH), luteinizing hormone (LH), Prolactin (PRL) and total testosterone were quantified by Chemiluminescent Microparticle ImmunoAssay (CMIA, Architect System; Abbott Laboratories, Abbott Park, IL, USA). Detection limits, intra- and inter-assay coefficients of variation and normal ranges were previously described [32]

### Sexual function (IIEF- 15 questionnaires)

Sexual function was evaluated through self-administered questionnaires as described elsewhere [34;35].

### Testicular ultrasonography (US)

Testicular US examinations were performed in both Rome and Latina centers using standardized views for testicular and epidydimal evaluation with 7–15 MHz wideband linear transducers as described in Pozza et al. 2020 [35]. Testicular volume was estimated using the formula for a prolate ellipsoid: length (L) x width (W) x height (H) × 0.52 [36].

### Statistical analysis

Continuous variables are presented as mean and standard deviations or median and interquartile range, based on data distribution as evaluated by Kolmogorov–Smirnov test. Differences between groups are evaluated by Mann Whitney *U* or Kruskal–Wallis test, as appropriate. Where multiple comparisons are performed, post-hoc results are adjusted according to the Bonferroni method. Categorical variables are presented as counts and percentages and are compared by χ^2^ test. Statistically significant correlations among the variables examined were evaluated using Spearman’s rank correlation test. For analyses, we grouped patients in two severity grades: “Severe/Critical” and “Mild/Moderate”. The probability values are 2-sided and a *p*-value < 0.05 was considered statistically significant. All computations were carried out with Statistical Package for the Social Sciences (SPSS) 25.0 (SPSS Inc., Chicago, USA).

## Results

Based on inclusion/exclusion criteria, we have been able to recruit 80 patients recovered from SARS-CoV-2 (45 subjects from Latina and 35 from Rome). Additionally, two control groups who were never positive to SARS-CoV-2 have been retrospectively selected: 98 normozoospermic subjects with no previous andrological pathologies (CTR1) and 98 infertile subjects (CTR2) who previously attended the Reproductive Medicine center for couple infertility of Policlinico Umberto I-Rome. The two populations of the cases group were comparable by age (Rome: 40.8 ± 13.1 years vs Latina 45.6 ± 10.0 years; *p* = 0.156) and BMI (Rome: 25.6 ± 3.9 vs Latina: 27.4 ± 4.3; *p* = 0.063). Table 1 shows relevant demographics and comorbidities of the investigated population. In particular, 38/80 subjects (47.5%) already had achieved fatherhood. Subject’s occupations are described in Supplementary Table 1. Furthermore, treatments listed in the medical records of each subject were quite heterogeneous and included the following drugs: hydroxychloroquine, lopinavir/ritonavir, darunavir/cobicistat, tocilizumab, azithromycin, paracetamol, ibuprofen, corticosteroids. According to COVID 19 severity patients were classified as: 32 patients Mild, 22pts Moderate, 15pts Severe and 11 pts Critical. Around eighty-eight percent of patients reported the presence of fever during the disease: in particular, the ten afebrile subjects all had a mild disease.

**Table 1:** Patients demographics

	Overall (80 pts)
Age	43.9 ± 11.7
BMI	26.6 ± 3.9
Cigarette Smoking	14%
Andrological Pathologies	17%
Sexological pathologies	23%
Hypertension	28%
Metabolical diseases	10%
Fatherhood	47.5%

Continuous variables are presented as means and standard deviations. Categorical variables are presented as percentages

### Semen parameters

**Fig. 1:**
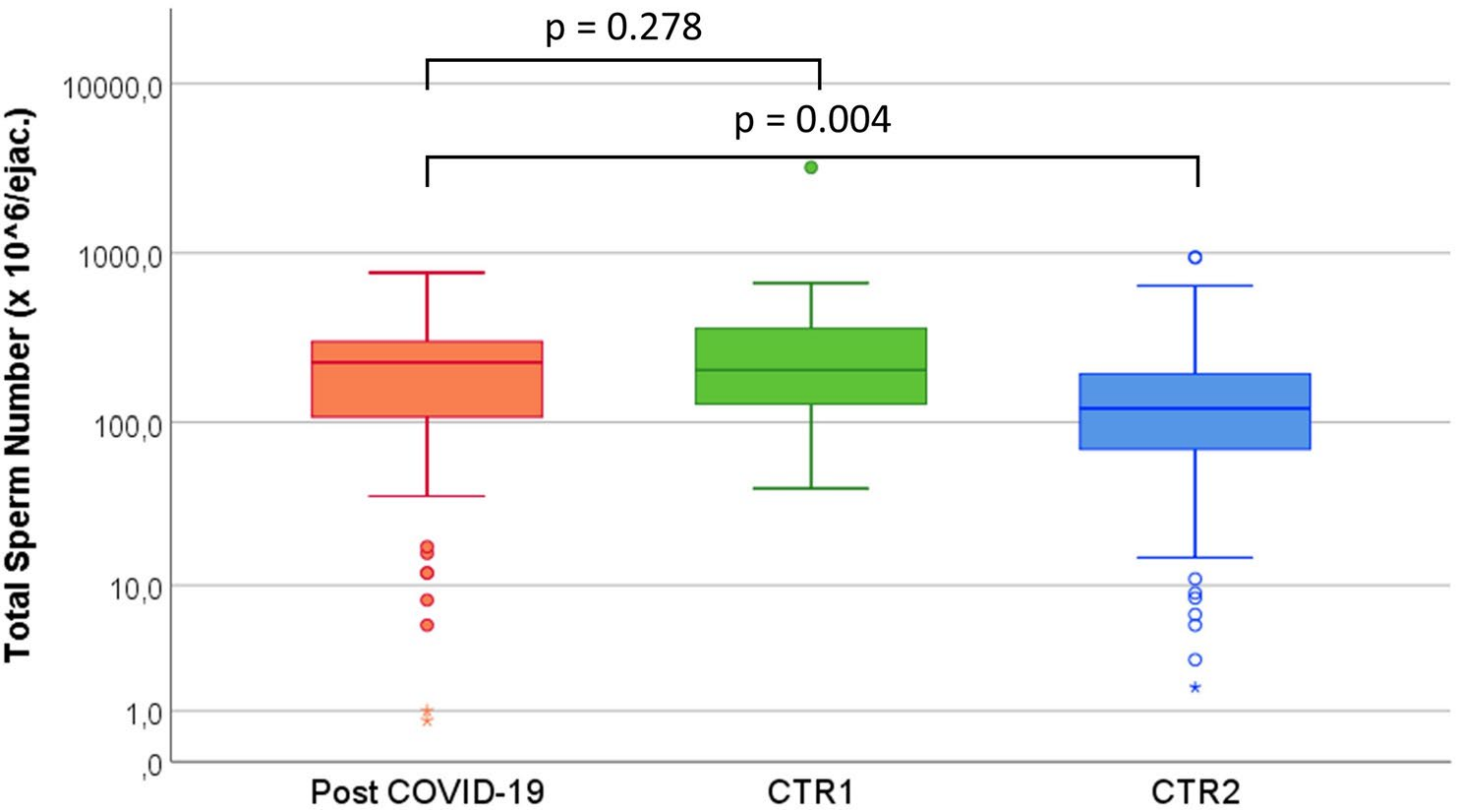
Comparison of total sperm number of SARS-CoV-2 recovered subjects (Post COVID-19) and Normozoospermic (CTR1) and Infertile (CTR2) subjects. (Kruskal–Wallis test, results are Bonferroni Adjusted for multiple comparisons)

**Fig. 2:**
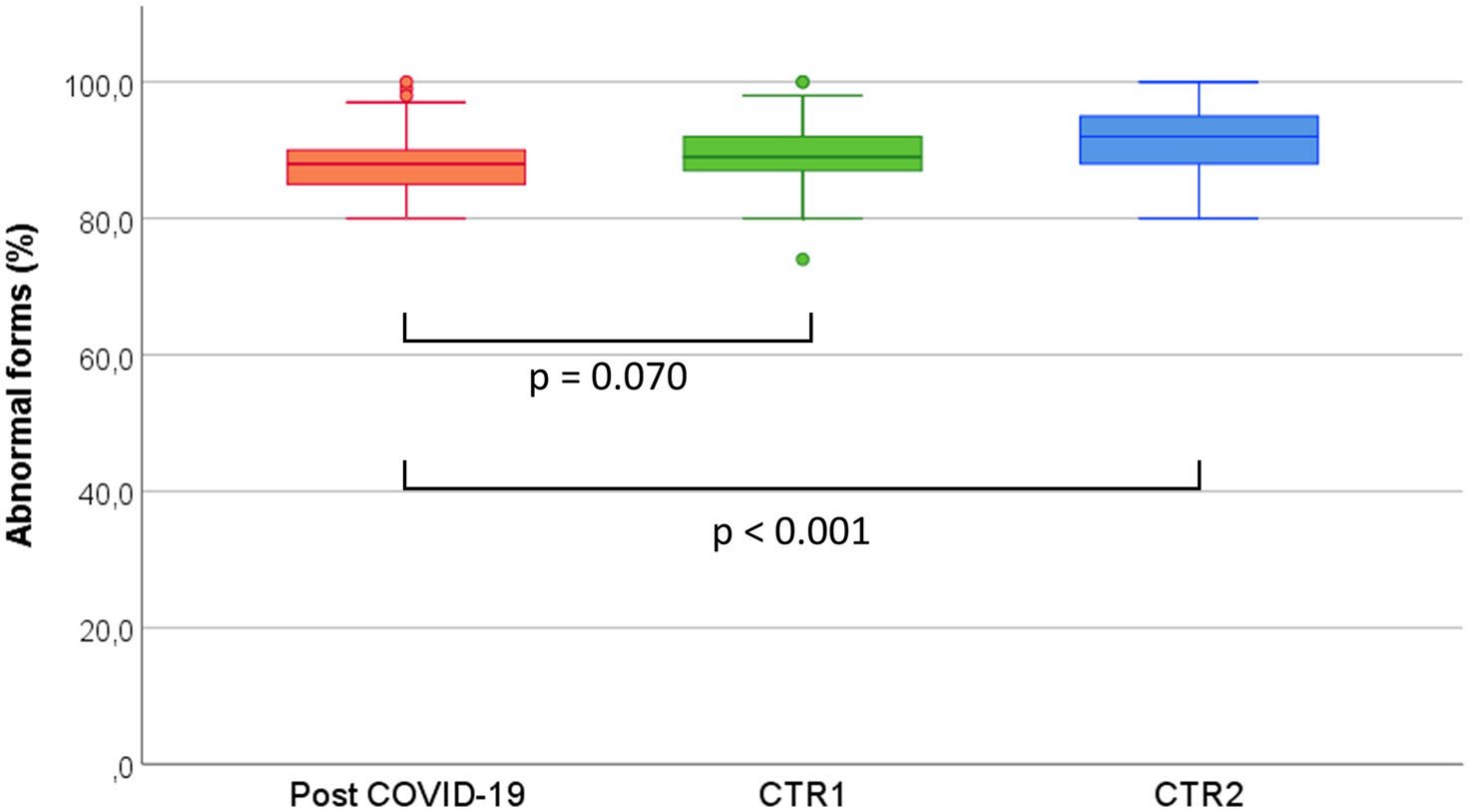
Comparison of abnormal forms (%) of SARS-CoV-2 recovered subjects (Post COVID-19) and Normozoospermic (CTR1) and Infertile (CTR2) subjects. (Kruskal–Wallis test, results are Bonferroni Adjusted for multiple comparisons)

Median of semen parameters from recovered SARS-CoV-2 subjects were within WHO 2010 fifth percentile. Sperm eosin vitality test showed that mean sperm viability was 63.8 ± 15.0%. No significant difference was found when comparing parameters from the normozoospermic control group (CTR1) (Table 2). In fact, total sperm number and percentage of abnormal forms between these two groups was comparable (*p* = 0.287 and *p* = 0.070, respectively), while these same parameters in post COVID-19 subjects were significantly better than infertile controls (CTR2) (*p* = 0.004 and *p* < 0.001, respectively) (Figs. 1, 2).

**Table 2:** Comparison of age and semen parameters of SARS-CoV-2 recovered subjects (cases) and Normozoospermic (CTR1) and Infertile (CTR2) subjects

	Volume (ml)	Sperm concentration (× 10^6^/ml)	Total Sperm Number (× 10^6^ /ejaculate)	Progressive Motility (%)	Abnormal Forms (%)
POST COVID-19 80 pz	3.1 ± 1.3 *3.0 *(2.0 –4.0)	72.6 ± 46.4 *72.0 *(38.0–96.0)	221.3 ± 151.8 *225.0 *(104.0–300.0)	40.6 ± 15.7 *45.0 *(30.0–5.0)	88.3 ± 4.3 *88.0 *(85.0–90.0)
CTR1 98 pz	3.3 ± 1.5 *3.0 *(2.0–4.2)	90.2 ± 90.1 *73.5 *(50.0–96.0)	278.6 ± 337.0 *202.8 *(127.4–357.5)	44.7 ± 12.7 *50.0 *(40.0–55.0)	89.8 ± 4.5 *89.0 *(87.0–92.0)
CTR2 98 pz	3.0 ± 1.7 *2.8 *(1.9–4.0)	60.6 ± 63.0^a^ *47.0 *(22.0–78.0)	158.0 ± 160.7^b^ *120.0 *(69.0–192.0)	38.9 ± 15.2 *45.0 *(30.0–50.0)	91.5 ± 4.3^c^ *92.0 (88.0*–*95.0)*
*P*-value	*0.381*	< *0.001*	< *0.001*	*0.017*	< *0.001*

(Means ± standard deviations, medians in italics and 25°–75° percentile in brackets) (Kruskal–Wallis test, post-hoc results are Bonferroni adjusted for multiple comparisons)

^a^*p* < 0.05 vs “post COVID-19” group

^b^*p* < 0.01 vs “post COVID-19” group

^c^*p* < 0.001 vs “post COVID-19” group

Moreover, while overall oligozoospermic subjects were 13/80 (16.2%), we could observe that when stratified for COVID-19 severity the prevalence of oligoozoospermia nearly doubles in severe cases (12.7% in mild subjects vs. 24.0% in severe subjects) although this does not reach statistical significance (Fisher Exact test *p* = 0.098).

**Fig. 3:**
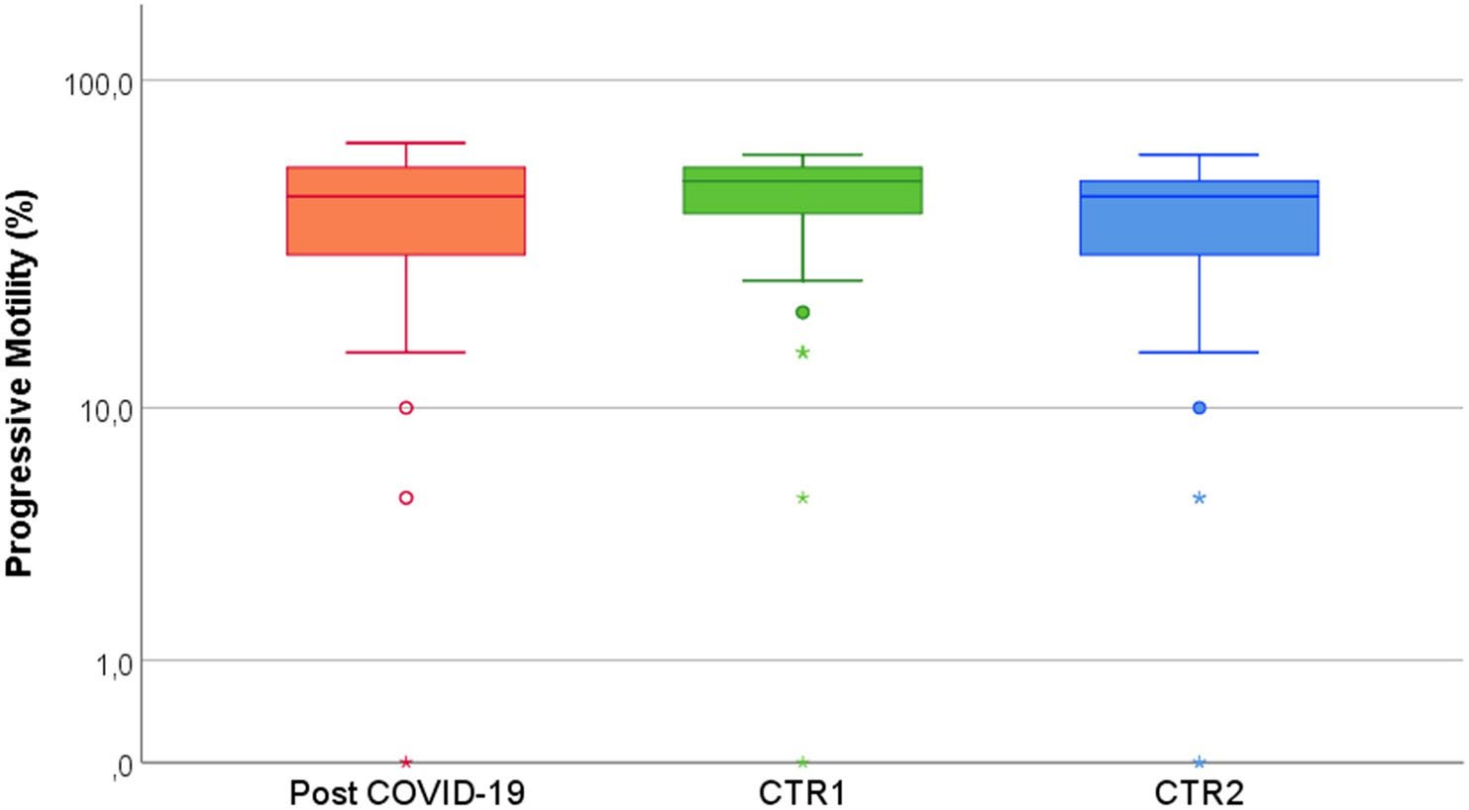
Comparison of progressive motility (%) of SARS-CoV-2 recovered subjects (Post COVID-19) and Normozoospermic (CTR1) and Infertile (CTR2) subjects. (Kruskal Wallis test, results are Bonferroni Adjusted for multiple comparisons)

Regarding progressive motility, we could not detect differences in semen samples from SARS-CoV-2 recovered subjects vs both Normozoospermic (CTR1) and Infertile controls (CTR2) (Table 2 and Fig. 3). Additionally, no significant association was detected between sperm parameters and both the previous presence of fever or COVID-19 severity at three months from recovery (data not shown).

### Antisperm antibodies (ASA) evaluation

The presence of ASA has been evaluated with both direct and indirect tests. Direct assays (SpermMar IgG and IgA) were performed in 62 subjects (18 samples were excluded due to oligoozoospermia or asthenozoospermia): only 1/62 subjects (1.6%) were found positive to IgG class. Indirect testing, Gelatin Agglutination Test (GAT), was performed in all subjects in both semen plasma and blood serum: we detected 3/77 (3.9%) positive blood serum samples, but no positive semen plasma sample (Supplementary Table 2). Of note, all subjects with positive blood serum samples had severely altered semen parameters and, because of this, direct tests could not have been performed in these subjects.

### Sperm DNA fragmentation

**Fig. 4:**
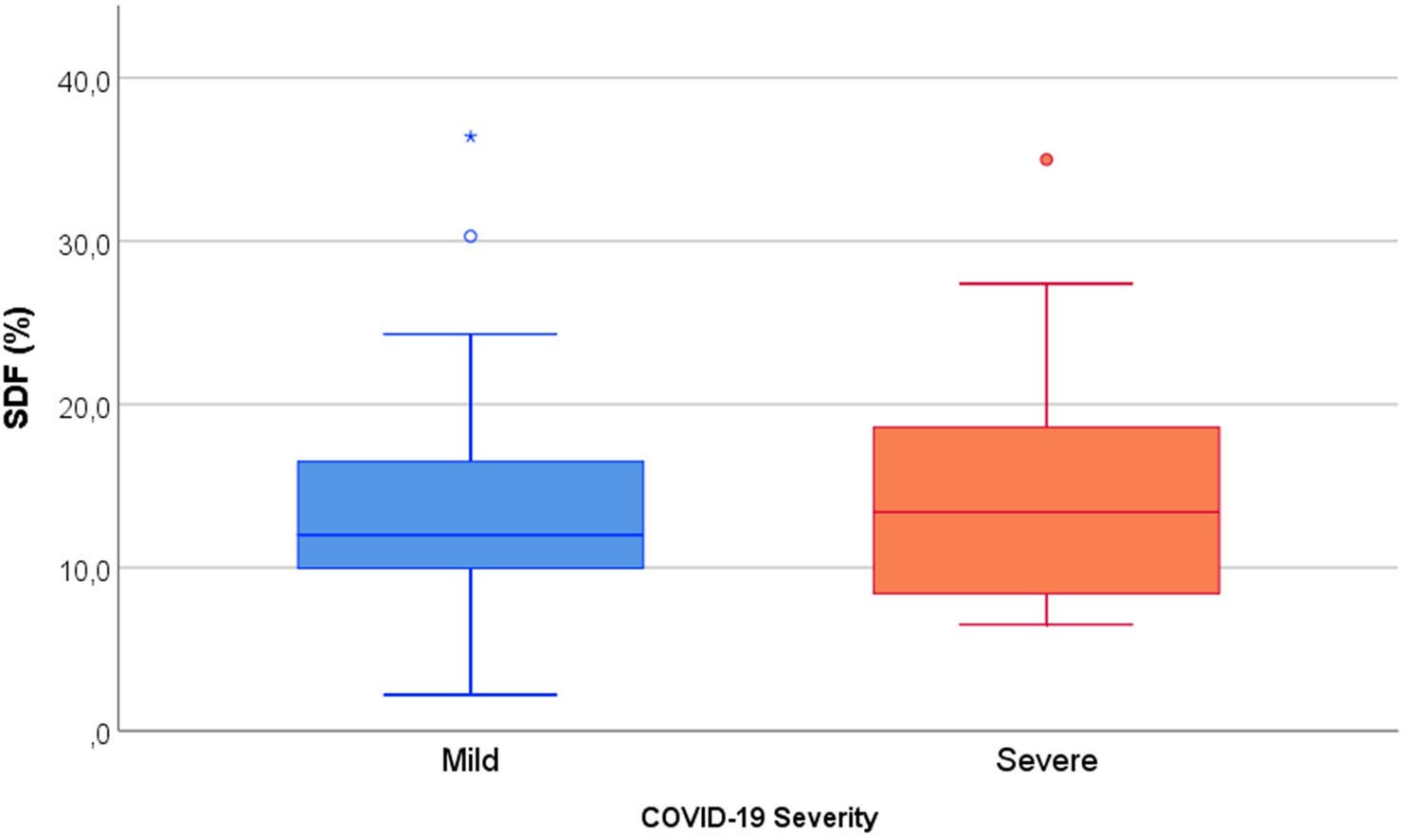
Comparison of SDF (%) of SARS-CoV-2 recovered subjects (Post COVID-19) stratified per COVID-19 severity groups. (Mann Whitney *U* test)

Chromatin integrity analysis showed that the mean percentage of sperm DNA fragmentation (%SDF) in SARS-CoV-2 recovered subjects was 14.1 ± 7.0% (median 12.4%). We compared this to a previously published normozoospermic control population, and we observed that both values were comparable (Carlini et al. 2017—12.8 ± 5.3%, median 12.2%) [37]. Additionally, %SDF did not differ significantly between COVID-19 severity groups (*p* = 0.538) (Fig. 4) or between subjects with/without fever (*p* = 0.939) but correlated significantly with patients’ age (*ρ* = 0.282; *p* = 0.031).

### Hormone profile

Table 3 shows the hormone profile of recruited COVID-19 recovered subjects. Remarkably, mean levels of investigated hormones (LH, FSH, total testosterone and prolactin) were well within normal ranges. We could detect that only five subjects (6.2%) had total testosterone levels below the laboratory reference range (< 10.4 nmol/l). The prevalence of biochemical hypogonadism was comparable between the two participating centers. Moreover, it should be stressed that testosterone levels did not differ significantly between COVID-19 severity groups (*p* = 0.423), and the pattern of testosterone levels among groups is shown in Supplementary Fig. 1.

**Table 3:** Hormone levels of SARS-CoV-2 recovered subjects (cases) in the whole caseload

	FSH (mUI/l)	LH (mUI/ml)	PRL (ng/dl)	Total testosterone	% TT below laboratory reference
Whole Caseload	4.7 ± 3.6 *3.9 *(2.8–5.6)	3.7 ± 2.0 *3.4 *(2.5–4.5)	10.4 ± 5.1 *9.8 *(7.0–12.6)	19.2 ± 8.1 *17.2 *(13.5–22.5)	6.2% 5/80

(Means ± standard deviations, medians in italics and 25°–75° percentile in brackets)

### Testicular ultrasonography

Testicular ultrasound evaluation of COVID-19 recovered subjects showed that all patients had normal testicular volume, ultrasound echotexture and echogenicity and, in general, all ultrasound findings were consistent with patients’ age. No subject showed ultrasound signs of testicular damage or suggestive of previous orchitis. Mean bilateral testicular volume was 31.5 ± 9.6 ml (median 30.9). In seven subjects, we could detect a unilateral left varicocele (grade I-II) in absence of significant testicular asymmetry.

### Sexual function—IIEF-15

Sexual function investigated through IIEF-15 and Table 4 shows the scores of the various domains of the questionnaire. Erectile dysfunction (Erectile function domain score < 26) was detected in 30% of subjects. Even though we did not find significant differences in IIEF-15 domains among COVID-19 severity scores (erectile function domain scores *p* = 0.473 Mild vs Severe), we could observe a trend of reduction of EF domain score in highest severity grades as well as a trend of increase in prevalence of erectile dysfunction (Supplementary Fig. 2).

**Table 4:** Summary of IIEF-15 domains from SARS-CoV-2 recovered subjects (cases) in the whole caseload

	Erectile function domain	Orgasmic function domain	Sexual desire domain	Intercourse satisfaction domain	General satisfaction domain	% Erectile dysfunction (EF domain < 26)
Whole Caseload 80 pts	24.4 ± 6.3 *27.0 *(23.0–29.0)	8.8 ± 1.8 *10.0 *(8.0–10.0)	7.8 ± 1.9 *8.0 *(7.0–9.0)	10.6 ± 2.8 *11.0 *(9.0–12.0)	7.2 ± 2.5 *8.0 *(5.0–10.0)	30.0% (24/80)

## Discussion

From December 2019, the COVID-19 became pandemic; and in the last two years, infected billions of people worldwide marking a still active planetary emergency. Up to February 2022 more than 12 millions of Italian cases have been reported with nearly 150 thousands COVID-19 deaths (website ISS EpiCentro, last accessed 22-02-2022). While clinical characteristics of the disease have slightly changed during the years due to the circulation of new variants and, above all, the intensive vaccination campaign, there is still great concern for infected patients. Attention has moved towards possible long-term consequences of the infection in terms of cardiovascular, pneumological, neurological and endocrine health. In particular, concerns for male reproductive health have been raised in terms both of direct and indirect testicular damage. Since the outbreak, epidemiological data of SARS-CoV-2 showed a higher incidence and severity in men. Despite a likely multi-factorial etiology, ACE2 and TMPRSS2 expression in the male genital tract might in part explain these earlier observations. Nonetheless, there is paucity if in vivo evidence of COVID-19 related orchitis. Furthermore, we recently reported that SARS-CoV-2 has a negligible chance of detection in semen [2], thus weakening the hypothesis of direct testicular damage.

This, however, does not exclude possible indirect effects on testicular function. COVID-19 pathophysiology includes a dysregulated immune response [38]. While cytokines are essential immune mediators to contrast the infection, their dysregulation might induce harmful consequences, and might also play a relevant role in the induction of testicular side effects mainly through stimulation of local inflammation and oxidative stress.

Recent illnesses, especially when the patient has reported fever or the utilization of certain drugs, are known to possibly affect the ejaculate (WHO 2021) and, thus, semen quality [39]. Antiviral drugs and immunomodulators are currently the mainstay of COVID-19 treatment, especially in moderate to severe cases, but in early days of the pandemic due to limited knowledge the use of heterogeneous drug protocols have been reported as it can also be noted in our caseload, where we registered a wide use of corticosteroids, antibiotics and hydroxychloroquine. Corticosteroids might alter testicular hormone axis and increase SHBG levels. Although evidence is generally of low quality, all these drugs are known to transiently affect semen parameters [40] and their negative effects on spermatogenesis might be synergistic with the indirect effects of fever and COVID-19 itself. Since these effects might endure for a full spermatogenic cycle, evidence of post COVID-19 alteration in semen parameters should be interpreted in regards of distance from recovery as it is likely that post infection semen quality might be transiently affected. Few papers have investigated post COVID-19 semen parameters, and can be roughly divided into those which have analysed semen parameters within a median of 30–40 days [7, 20–22, 41] (Table 5a) and those who reported data on recovered subject from more than 60 days [13, 23, 24, 26, 27] (Table 5b). Short term data show a wide range of semen outcomes, from a higher incidence of azoospermia to normozoospermia (Table 5a). Holtmann et al. [20] and Guo et al. [41] reported that recovered patients had semen parameters within WHO 2010 5^th^ percentile, but it must be remarked that most subject recruited had only a mild disease. On the other hand, Gacci et al. [22] reported a prevalence of 18.6% of azoospermia and 7.0% of severe oligoasthenoteratozoospermia, and a correlation between azoospermia and COVID-19 severity. However, in this study patients with more severe disease were also enrolled, and this might have had a direct impact on reported results. Finally, when comparing COVID-19 recovered patient to healthy controls, recovered subjects were reported to have worse semen parameters [7, 21]. Overall, these studies reported cases with a median recovery from around one month and the lack of pre-COVID semen analyses and longer follow-up do not allow to exclude that patients with severe alterations of spermatogenesis either had preexisting damage to spermatogenesis or have only a transient damage. In this sense, Hajizadeh Maleki and Tartibian [7] also reported a higher %SDF in patients closer to recovery, which improves during follow-up (up to 60 days). The impact of fever associated with SARS-CoV-2 (present in up to 88% of our infected cases) was only reported in two studies, but with contrasting results (Table 5a). Transient effects of fever on semen quality and sperm DNA integrity have already been described [17, 18, 42]. It has been observed that semen parameters return comparable to baseline 79 days after the fever [18]. Moreover, Evenson et al. (2000) studied sperm chromatin structural integrity in a fertile man who contracted influenza, demonstrating an altered chromatin structure 18–66 days post-fever that returned to normal value near the completion of the spermatogenesis (74 days) [43]. Short delay after recovery in most studies probably does not allow to ascertain real effects of the infection on spermatogenesis and limiting the seminological assessment to semen analysis further limits the evaluation. Erbay et al. [23], in particular, showed that a caseload of 69 patients had worse semen parameters more than 90 days after disease recovery. The strength of this study is the presence of pre-COVID-19 semen analyses, but all patients were selected from an infertility clinic and could possibly represent a subgroup of population of infertile subjects whose spermatogenesis was more vulnerable to the direct/indirect effects of the virus. Other studies, however, did not confirm these findings [13, 24]. Donders et al. [27], provided a thorough seminological evaluation of 118 patients, including sperm DNA integrity and ASA evaluation. While incidence of ASA was only 3/119 subjects, semen parameters and %SDF was significantly lower close to recovery and progressively improved returning to normal after three months from recovery. Results from Ruan et al. [26] confirmed the absence of %SDF alterations over 3 months from recovery but semen parameters were nonetheless worse than healthy controls, in absence of significant hormone alterations. Furthermore, testicular US parameters appeared well within the normal values detected in fertile patients [44].

**Table 5:** Currently published papers with focus on post COVID-19 semen parameters which provided semen analyses within 60 days **a** and over 60 days **b**

References	Caseload (pts)	Days since recovery Median (range)	COVID-19 Severity	Fever	Hormonal evaluation	Sperm DNA integrity evaluation	ASA evaluation	Erectile funtion evaluation	Results
(a)									
Holtmann et al. [20]	18 cases 14 controls	32,7 (8–54)	Mild 14 pts; moderate 4 pts	10/18 (55,5%) pts	NO	NO	NO	NO	Semen parameters within WHO 2010 5th percentile; moderate patients and those with fever had worse semen parameters
Li et al. [21]	23 cases 22 controls	26,0 (4–42)	Mild 9 pts; hospitalized 14pts	Fever < 39 °C or no fever in 15/23 pts; fever ≥ 39 °C in 8/23 pts	NO	NO	NO	NO	Sperm concentration reduced in cases; no difference between severity or fever groups
Gacci et al. [22]	43 cases	n/a (24–43)	12 home treatment; 31 hospitalized (of whom 5/31 in intensive care unit)	n/a	NO	NO	NO	YES	8/43 azoospermic and 3/43 severe OAT; prevalence of azoospermia correlates with reported COVID19 severity
Guo et al. [41]	23 cases	32,0 (26–34)	Mild 18 pts; moderate 5 pts	n/a	NO	NO	NO	NO	Semen parameters within normal ranges
Maleki and Tartibian [7]	84 cases 105 controls	n/a (10–60)	Mild 1 pt; moderate 23 pts; severe 27 pts; critical 33 pts	n/a	NO	YES	NO	NO	Sperm concentration, sperm motility and SDF% were worse in cases than controls and improved during the follow up
(b)									
Donders et al. [27]	118 cases	< 31,0 (n/a) in 35 pts; n/a (32–62) in 51 pts; > 63 days (n/a) in 32 pts	23,7% of subject > 5 symptoms; 5/118 (4,25%) hospitalized (of whom 2 in intensive care units)	N/A	NO	YES	YES	NO	Semen quality and SDF worse close to recovery and normal > 63 days. No correlation with COVID severity
Ma et al. [13]	12 cases (semen analysis)	78,0 (56–109)	Mild 1 pt; moderate 11 pts	N/A	YES	YES	NO	YES (not shown)	4/12 of cases with low motility and high SDF%
Gul et al. [24]	29 cases	n/a (90–240)	3/29 pts pneumological signs 26/29 home treatment	N/A	YES	NO	NO	NO	No difference in pre vs post COVID19 semen parameters
Erbay et al. [23]	69 cases from infertility clinic	Mild pts: n/a (94–144) moderate pts: n/a (96–190)	Mild 26 pts; moderate 43 pts	N/A	NO	NO	NO	NO	mild pts: sperm motility and viability worsened post COVID19 vs pre COVID19; moderate pts: all semen parameters worsened after COVID19
Ruan et al. [26]	55 cases and 145 controls	80,0 (64–93)	Mild 7 pts; moderate 24 pts; severe 24 pts	N/A	YES	YES	NO	NO	Worse total sperm number and motility in cases vs controls. Sperm concentration was found correlated with disease severity. No alterations in SDF% detected both within and over 90 days from recovery
Present study	80 cases 96 normazoospermic controls 96 infertile controls	90 days	32 pts Mild, 22pts Moderate, 15pts Severe and 11 pts Critical	64/80 (80%) pts	YES	YES	YES	YES	No significant alteration of investigated parameters

Our results showed that overall andrological health appears not to be compromised 3 months after COVID-19 recovery. In particular, it can be assumed that after a full spermatogenetic cycle from recovery semen parameters and SDF% present no significant long-term impairment and no sperm autoimmune response has taken place. Likewise, hormone profile did not show relevant alterations. To further support these findings, the ultrasound study did not show the presence of any testicular parenchymal damage. Remarkably, investigated parameters were not significantly associated with severity of COVID-19, further strengthening the hypothesis that, once clinical recovery has taken place, SARS-CoV-2 is unlikely to be causative of potential alterations of andrological parameters.

We did find, however, the presence of erectile dysfunction in roughly one-third of subjects. This aspect of post-COVID andrological health aims to be purely descriptive of a relatively under-investigated issue that will undoubtedly require further specific investigations. This first post COVID-19 screening of sexual functioning may be an important point to remark, as it may represent a long-term effect of drugs and/or COVID-19 related distress and may impact on both reproductive health and quality of life [45–49]. Recent reports highlighted the association of SARS-CoV-2 infection and increased prevalence of ED [50, 51]. Sivitrepe et al. investigated the presence of ED after three months of hospital discharge for COVID-19 detecting a further worsening of IIEF scores compared to the scores at hospital admission, linking this worsening to IL-6 levels [52]. Unfortunately, the recruited subject also had high glycemic levels, suggesting diabetes and cardiometabolic comorbidities as possible confounders. In our caseload, despite the lack of pre-COVID IIEF scores, the prevalence of ED appears age-dependent in absence of symptomatic metabolic diseases. Therefore, a viral-induced inflammatory state and endothelial dysfunction might suggest some degree of COVID-19 contribution to the ED in the recovery phase [53], but it is also likely that this might be associated to underlying metabolic disorders.

It is our opinion that the investigation of post COVID-19 sexual functioning might reveal those subjects who will likely require a more careful andrological follow-up in the possibility of post-COVID persistent effects, but this requires more in-depth analysis in future studies.

Strengths of the present study are the complete andrological evaluation of patients and their recruitment from a general population of SARS-CoV-2 infected subjects from Infectious Diseases Departments, at least partially overcoming selection biases from other studies recruiting an infertile population. Furthermore, the inclusion of sexological evaluation, testicular ultrasonography, hormone profile, ASA and sperm DNA integrity evaluation in the work up of post COVID-19 patients allowed to perform a comprehensive andrological evaluation of these subjects. A possible limitation to generalization is the absence of pre-infection data, but comparisons with both infertile and normozoospermic pre-COVID-19 subjects more than compensate for this limitation.

## Conclusions

Post COVID-19 subjects appear as a possibly vulnerable population to long-term systemic effects of the infection. Nonetheless, our data further remark that the virus does not seem to cause direct damage to the testicular function, while indirect damage due to inflammation, drugs and fever appear to be transient. This provides a strong and reassuring indication to couples that are attempting to conceive either naturally or artificially. Nonetheless, a careful monitoring of these subjects appears necessary [54]. In particular, due to evidence of transient alterations of sperm DNA integrity close to the recovery, it is possible to counsel infertile couples to postpone the research of parenthood or ART procedures around three months after recovery from the infection to maximize their reproductive chances.

## Supplementary Information

Below is the link to the electronic supplementary material.

Supplementary file1 (JPG 231 KB) Supplementary Fig. 1 Comparison of total testosterone (nmol/l) levels of SARS-CoV-2 recovered subjects stratified per COVID-19 Severity. (Mann Whitney U test)

Supplementary file2 (JPG 172 KB) Supplementary Fig. 2 Comparison of Erectile Function domain scores of SARS-CoV-2 recovered subjects stratified per COVID-19 Severity. (Mann Whitney U test)

Supplementary file3 (DOCX 14 KB) Supplementary Table 1 Occupations of recruited subjects

Supplementary file4 (DOCX 13 KB) Supplementary Table 2 Summary of ASA positive subjects. “*” indicates that direct testing could not be performed due to severe oligoasthenoteratozoospermia (OAT)

## Data Availability

The data underlying this article will be shared on reasonable request to the corresponding author.
