# Supplementary figures and images for: Male reproductive health after 3 months from SARS-CoV-2 infection: a multicentric study
Source: J Endocrinol Invest. 2022 Aug 9;46(1):89–101. doi: 10.1007/s40618-022-01887-3 (PMC9362397; doi:10.1007/s40618-022-01887-3)

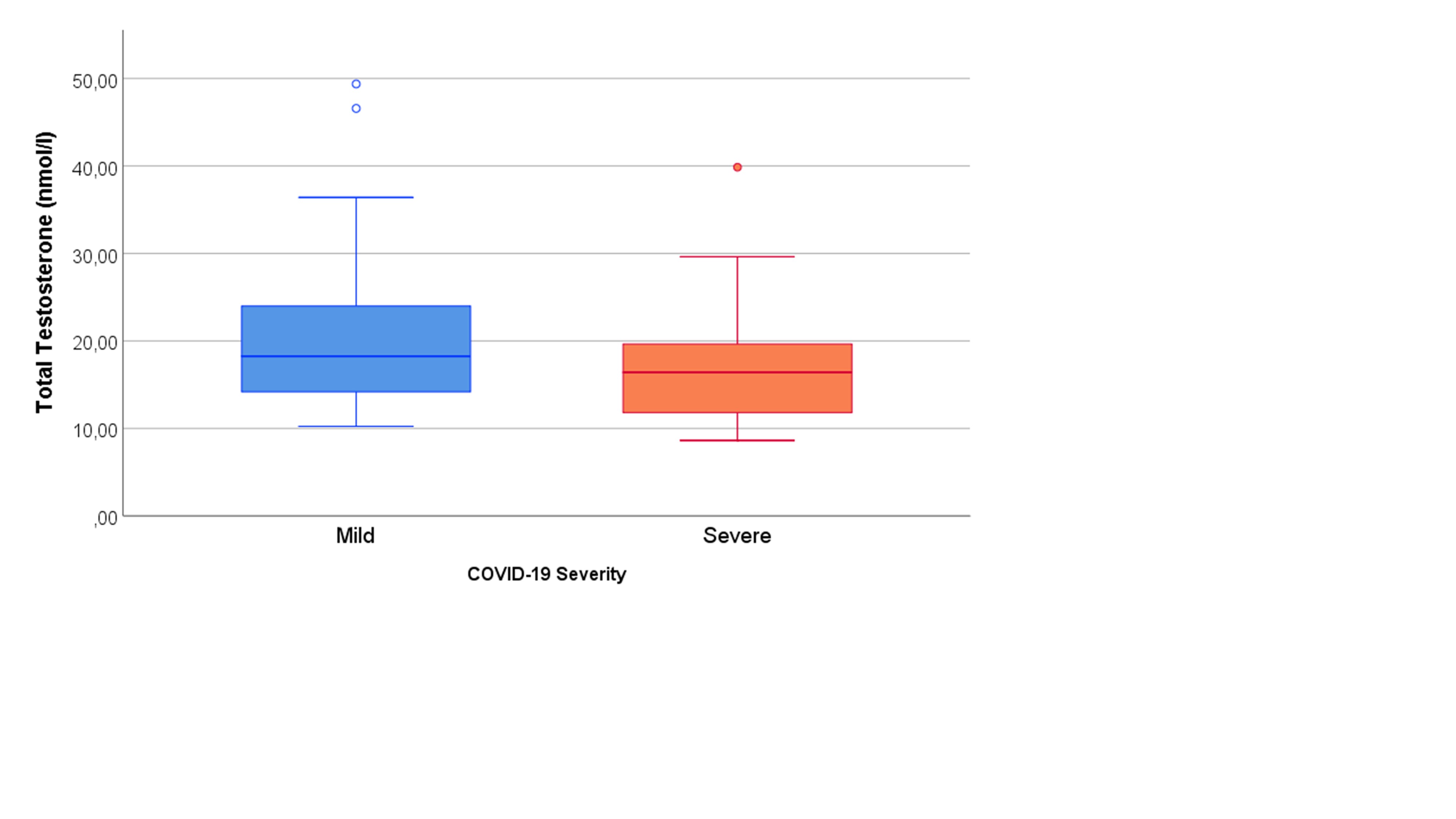

Supplement: Supplementary file 1 — Supplementary file1 (JPG 231 KB) Supplementary Fig. 1 Comparison of total testosterone (nmol/l) levels of SARS-CoV-2 recovered subjects stratified per COVID-19 Severity. (Mann Whitney U test) [file 40618_2022_1887_MOESM1_ESM.jpg]

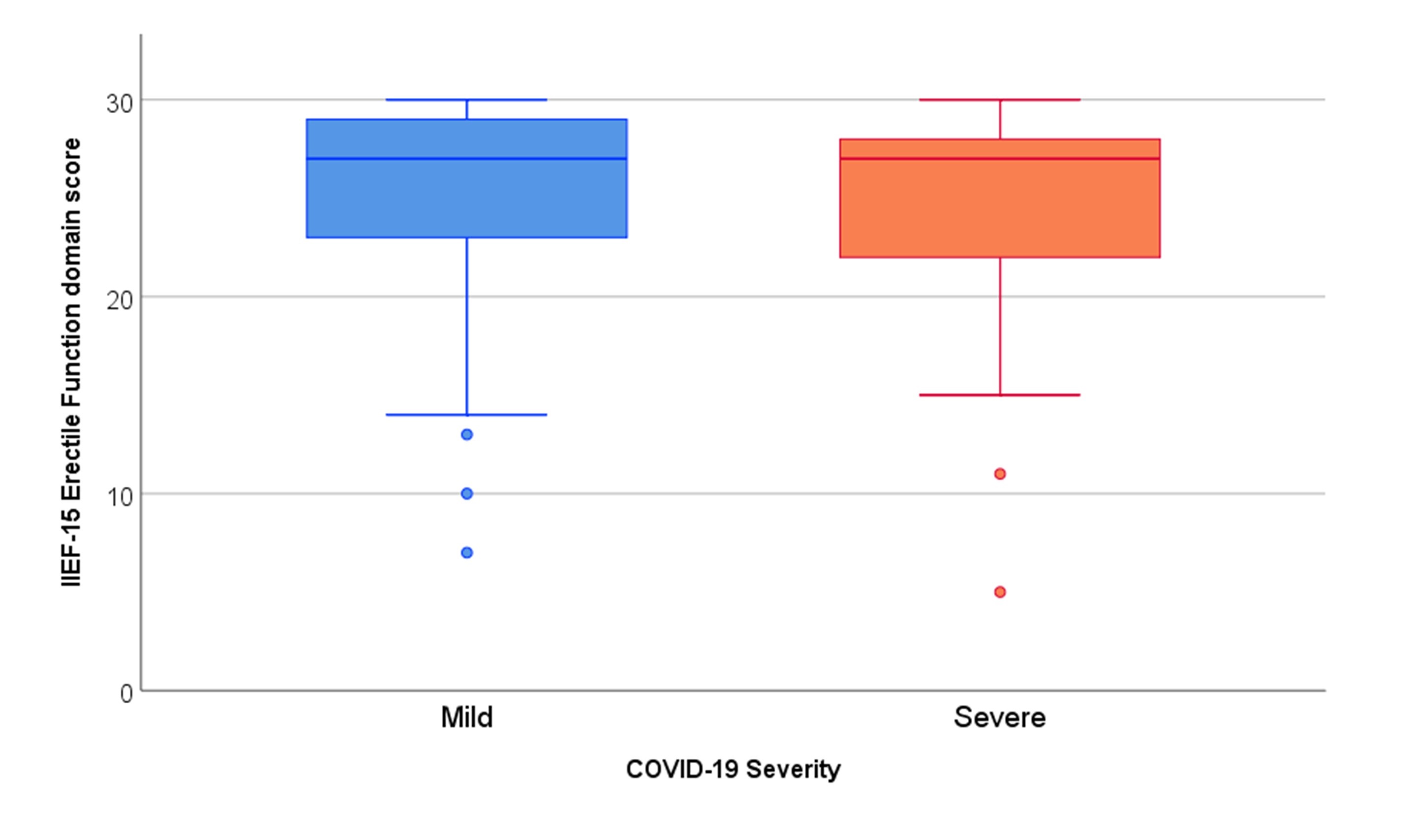

Supplement: Supplementary file 2 — Supplementary file2 (JPG 172 KB) Supplementary Fig. 2 Comparison of Erectile Function domain scores of SARS-CoV-2 recovered subjects stratified per COVID-19 Severity. (Mann Whitney U test) [file 40618_2022_1887_MOESM2_ESM.jpg]
