# Supplementary material for: Male reproductive health after 3 months from SARS-CoV-2 infection: a multicentric study
Source: J Endocrinol Invest. 2022 Aug 9;46(1):89–101. doi: 10.1007/s40618-022-01887-3 (PMC9362397; doi:10.1007/s40618-022-01887-3)
Supplement: Supplementary file 3 — Supplementary file3 (DOCX 14 KB) Supplementary Table 1 Occupations of recruited subjects [file 40618_2022_1887_MOESM3_ESM.docx]

| **Subjects Occupation** | | |
| --- | --- | --- |
| Office worker | 19 | 23.8% |
| Heavy/Factory worker | 13 | 16.3% |
| Student | 9 | 11.3% |
| Freelance | 7 | 8.8% |
| Healthcare services | 6 | 7.5% |
| Military | 5 | 6.2% |
| Manager | 4 | 5.0% |
| Driver | 3 | 3.7% |
| Door-to-door salesman | 3 | 3.7% |
| Teacher | 2 | 2.5% |
| Cook | 2 | 2.5% |
| Retired | 1 | 1.2% |
| Unemployed | 6 | 7.5% |

**Supplementary Table 1 –** Occupations of recruited subjects.
