# Supplementary material for: Male reproductive health after 3 months from SARS-CoV-2 infection: a multicentric study
Source: J Endocrinol Invest. 2022 Aug 9;46(1):89–101. doi: 10.1007/s40618-022-01887-3 (PMC9362397; doi:10.1007/s40618-022-01887-3)
Supplement: Supplementary file 4 — Supplementary file4 (DOCX 13 KB) Supplementary Table 2 Summary of ASA positive subjects. “*” indicates that direct testing could not be performed due to severe oligoasthenoteratozoospermia (OAT) [file 40618_2022_1887_MOESM4_ESM.docx]

| **Patient** | **Semen sample** | **SpermMar IgG** | **SpermMar IgA** | **GAT blood serum** | **GAT seminal plasma** |
| --- | --- | --- | --- | --- | --- |
| #13 | Normozoospermic | 35% (tail) | Negative | Negative | Negative |
| #29 | OAT | * | * | 1:1024 | Negative |
| #58 | OAT | * | * | 1:4 | Negative |
| #79 | OAT | * | * | 1:256 | Negative |

**Table 3 –** Summary of ASA positive subjects. “*” indicates that direct testing could not be performed due to severe Oligoasthenoteratozoospermia.
